# A biomedical Engineering Laboratory module for exploring involuntary muscle reflexes using Electromyography

**DOI:** 10.1186/s13036-020-00248-z

**Published:** 2020-11-09

**Authors:** Karly S. Franz, Kramay Patel, Dawn M. Kilkenny

**Affiliations:** 1 Institute of Biomedical Engineering, University of Toronto, 164 College St Room 407, Toronto, ON M5S 3G9 Canada; 2 Bloorview Research Institute, Holland Bloorview Kids Rehabilitation, 150 Kilgour Rd, East York, ON M4G 1R8 Canada; 3 Krembil Research Institute, 60 Leonard Avenue, Toronto, ON M5T 0S8 Canada; 4 Faculty of Medicine, University of Toronto, 1 King’s College Circle, Toronto, ON M5S 1A8 Canada; 5 Institute for Studies in Transdisciplinary Engineering Education & Practice, University of Toronto, 35 St. George Street, Toronto, ON M5S 1A4 Canada

**Keywords:** Electromyography, Biomedical engineering, Undergraduate physiology, Laboratory protocol, TENS, Electrical stimulation, Stretch reflex

## Abstract

**Background:**

Undergraduate biomedical engineering (BME) students interested in pursuing a career in research and development of medical or physiological monitoring devices require a strong foundation in biosignal analysis as well as physiological theory. Applied learning approaches are reported to be effective for reinforcing physiological coursework; therefore, we propose a new laboratory protocol for BME undergraduate physiology courses that integrates both neural engineering and physiological concepts to explore involuntary skeletal muscle reflexes. The protocol consists of two sections: the first focuses on recruiting soleus motor units through transcutaneous electrical nerve stimulation (TENS), while the second focuses on exploring the natural stretch reflex with and without the Jendrassik maneuver. In this case study, third-year biomedical engineering students collected electromyographic (EMG) activity of skeletal muscle contractions in response to peripheral nerve stimulation using a BioRadio Wireless Physiology Monitor system and analyzed the corresponding signal parameters (latency and amplitude) using the MATLAB platform.

**Results/protocol validation:**

Electrical tibial nerve stimulation successfully recruited M-waves in all 8 student participants and F-waves in three student participants. The students used this data to learn about orthodromic and antidromic motor fiber activation as well as estimate the neural response latency and amplitude. With the stretch reflex, students were able to collect distinct signals corresponding to the tendon strike and motor response. From this, they were able to estimate the sensorimotor conduction velocity. Additionally, a significant increase in the stretch reflex EMG amplitude response was observed when using the Jendrassik maneuver during the knee-jerk response. A student exit survey on the laboratory experience reported that the class found the module engaging and helpful for reinforcing physiological course concepts.

**Conclusion:**

This newly developed protocol not only allows BME students to explore physiological responses using natural and electrically-induced involuntary reflexes, but demonstrates that budget-friendly commercially available devices are capable of eliciting and measuring involuntary reflexes in an engaging manner. Despite some limitations caused by the equipment and students’ lack of signal processing experience, this new laboratory protocol provides a robust framework for integrating engineering and physiology in an applied approach for BME students to learn about involuntary reflexes, neurophysiology, and neural engineering.

**Supplementary Information:**

The online version contains supplementary material available at 10.1186/s13036-020-00248-z.

## Introduction

Despite diversity across different undergraduate biomedical engineering (BME) curricula, most programs include physiology as a core part of the student learning experience [1] upon recognition of its importance in promoting interdisciplinary engagement [2]. Beyond understanding fundamental anatomical and physiological concepts, it is important for BME students interested in pursuing graduate education or a professional career designing and developing medical devices to learn effective acquisition of biosignals [3], because these responses can be monitored and measured to provide real-time feedback regarding system function.

Growth of applications in commercial, industrial, medical, and defense sectors has led to an increased demand in graduates well-versed in biological signal physiology, acquisition, interpretation, and application [4]. Therefore, it is critical to provide opportunities for our students to develop skills in biosignal data acquisition and analysis, with strong ability to translate outcomes to physiological function. Applied laboratory exercises for scientific education are widely documented to enhance learning because they allow students to explore theory within a practical, concrete context [5] and allow translation of concepts and development of professional skills [6]. Unfortunately, the dominant mode of curriculum delivery across BME programs remains in lecture format [1], likely due to time and budgetary constraints in relevant programs. Unfortunately, in the context of biosignal study, lack of practical experience restricts the opportunity for BME students to merge data outcomes with physiological concepts and translate them into tangible applications.

Because active learning approaches have been found to be particularly effective for teaching physiological concepts [7] meanwhile encouraging students to think at a higher level and engage in enhanced discussion with instructors [7], we are motivated to develop and implement economical solutions and include active learning approaches in our curriculum that focuses on physiological organ system integration. The sensorimotor system and its relationship with skeletal muscle reflexes is often included in introductory physiology course curricula because it provides an excellent model of physiological integration. During our course module on neurophysiology, students explored relevant concepts through the lens of engineering by using different approaches to evoke and analyze involuntary reflexes in skeletal muscles of the lower extremity. In addition to accomplishing this task using affordable, commercially available equipment, students correlated their own electrical biosignals to mechanical activity, observed physiological variability in the acquired signals across the class population, applied engineering skills by processing the signals for enhanced analysis, and translated data outcomes to lecture concepts through continued discussion.

Sensorimotor reflexes can be involuntary and are evoked naturally, through the stretch reflex, or artificially using an electrical stimulation. Additionally, application of electrical stimuli to induce physiological responses is a prominent area of BME research that is of strong interest to our students. Electrical stimulation can be used to restore lost function via functional electrical stimulation (FES), for rehabilitative purposes, or for muscle physiotherapy using neuromuscular electrical stimulation (NMES) [8–10]. While electrical stimulation applications are vast and can be implemented using a multitude of approaches, the fundamental concept is to deliver electrical pulses to the body to trigger compound action potentials in nerve fibers [8, 9]. Transcutaneous electrical nerve stimulation (TENS) is a cost-effective approach that is used in several applications to non-invasively interface with the peripheral nervous system [11]. In this way, TENS can be used to evoke involuntary motor responses through electrical stimulation. Depending on the stimulation amplitude and an individual’s nerve activation threshold, motor neurons can be recruited by stimulating sensory neurons that initiate a response that is similar to the natural stretch reflex known as the Hoffman Reflex [12]. At higher stimulation amplitudes, direct motor unit activation manifests as short latency M-waves while supramaximal stimulation causes antidromic motor neuron activation resulting in large-latency F-waves in the EMG signal.

Involuntary reflexes also occur naturally within the body. These natural stretch reflexes are spinal mechanisms induced by sensory stimuli that lead to skeletal muscle contractions. Like the H-reflex, stretch reflexes are a spinal mechanism that involve the entire sensorimotor loop and are commonly used in clinical settings to assess upper and lower neuron function [13]. This naturally occurring reflex can be reinforced and amplified using the Jendrassik Maneuver which is when an individual voluntarily clenches their teeth and pulls on their interlocked fists before the tendon-tap [13].

This newly developed laboratory experience explores the use of EMG to detect involuntary motor reflexes evoked by TENS, and also the knee-jerk reflex when occurring naturally or amplified using the Jendrassik Maneuver. The laboratory exercise was developed with three main objectives: 1) to support student learning of physiological course concepts; 2) to teach students how to use a wireless physiological monitoring system (the BioRadio; Great Lakes Neurotechnologies, Cleveland, OH) in conjunction with MATLAB (MathWorks, Natick, MA) to acquire and analyze relevant biosignals; and 3) to create a satisfying experience that promotes self-perception of the individual’s engineering skills. The described protocol demonstrates the opportunity for students to directly explore relevant physiological and basic electrical interfacing concepts related to the lower extremities using multiple analytical platforms, and demonstrates that physiological responses previously described can be elicited and measured using budget-friendly commercially available devices. Survey response feedback indicated that most of the students felt this lab protocol was an engaging approach to immerse in learning neurophysiology while applying their engineering knowledge. This newly developed protocol integrates neurophysiology and engineering concepts to provide a richer, more impactful experience for BME students to learn about involuntary physiological reflexes by exploring different approaches to evoke and analyze involuntary skeletal muscle reflexes.

## Methods

### Participants

Twenty-four third-year undergraduate students in a BME program at the University of Toronto participated in piloting the described laboratory protocol for an introductory physiology course. Students were divided into 8 groups (three students per group) and measured EMG signals from one volunteer within each group. This protocol was approved by the Research Ethics Board at the University of Toronto (REB #37563).

### Biosignal acquisition

#### Part 1: muscle recruitment using TENS

**Fig. 1:**
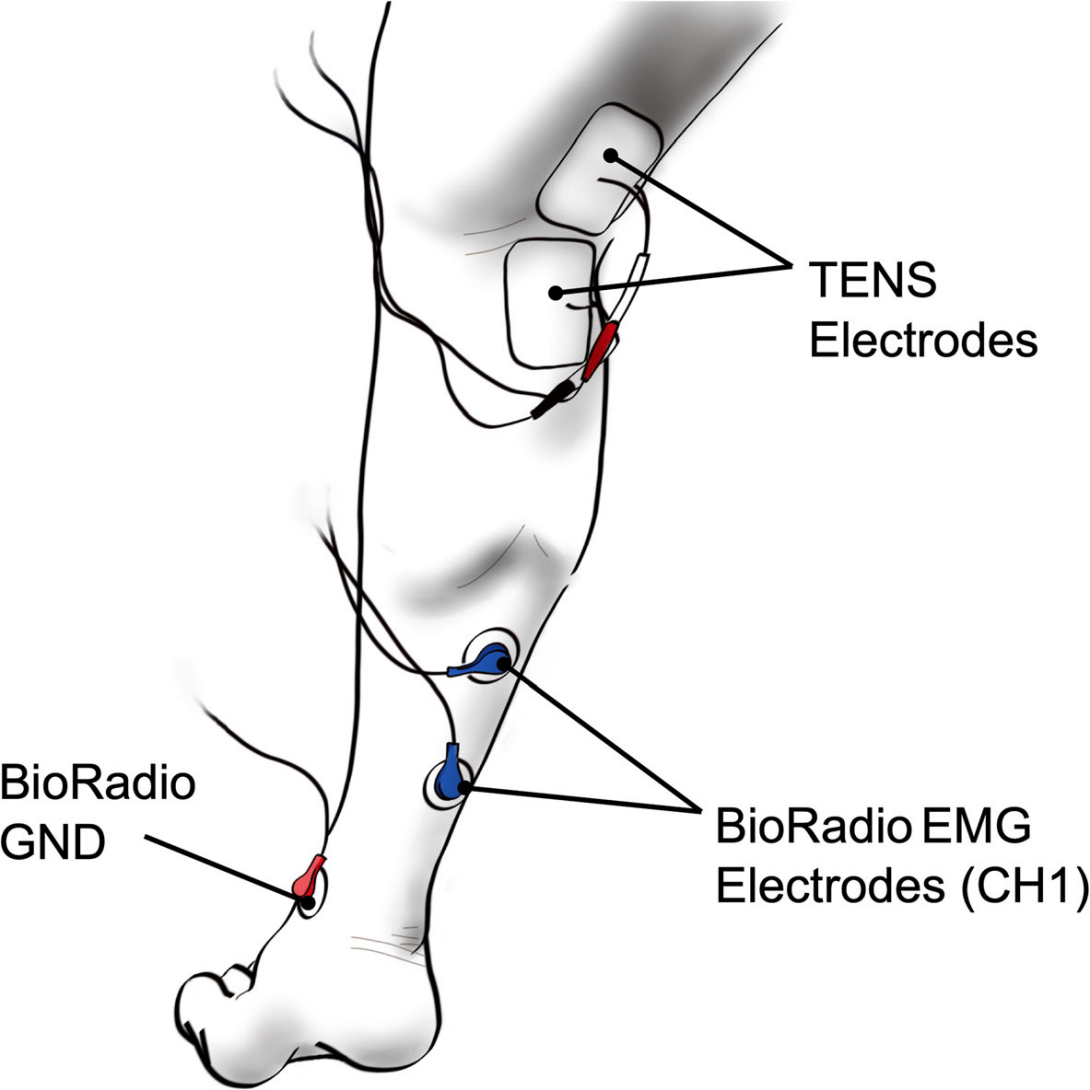
Placement of Simulating and Recording Electrodes. Two TENS stimulating electrodes are placed vertically along the superficial tibial nerve in the popliteal space. The soleus EMG activity was measured with sensing and reference electrodes aligning with the muscle fibers and connected to the positive and negative channel 1 (CH1) terminals of the BioRadio. The EMG signal was grounded with a third electrode placed on the medial malleolus

Soleus EMG activity was measured using the BioRadio (Great Lakes Neuro Technologies, Cleveland) and collected using the compatible BioCapture Research System software (version 5.5.640). The skin covering the soleus muscle was first cleaned using an alcohol wipe to prepare for electrode application (Covidien Kendall mini foam electrode, diameter 3 cm). Two electrodes, the sensing and reference, were subsequently placed on the cleaned area and connected to a single channel on the BioRadio (Fig. 1). The signal was grounded by placing the third electrode on the medial malleolus. Prior to placing the TENS electrodes on the legs, students verified that an EMG signal was detected by voluntarily contracting the soleus muscle multiple times and observing a corresponding increase in signal amplitude on the live BioCapture recording.

Once the soleus EMG activity was observed, students set up the TENS device (Classic TENS Unit, Body Clock, London) to stimulate the tibial nerve. Two square TENS electrodes (50 × 50 mm) were placed vertically on the popliteal fossa with the cathode connected proximally and the anode distally along the leg (Fig. 1). Continuous biphasic stimulation at 2 Hz with a 250 μs pulse width and an intensity between 1 and 80 mA was used to activate sensory and motor fibers of the tibial nerve. Students were instructed to gradually increase the stimulation intensity and take note of the amplitude at three different thresholds: sensory, motor, and maximum tolerable. The sensory threshold was defined as the minimum stimulation amplitude that caused electrical stimulus sensation with no motor activity. The motor threshold was defined as the minimum intensity needed to elicit an observable muscle twitch and the maximum tolerable was the highest stimulation amplitude that the student could apply without pain. Instructors emphasized that the activation thresholds would be different for each student and that correct neural recruitment should be painless.

After verifying that both the TENS and the BioRadio were properly set up, students began data collection. Students were instructed to record 5–7 stimulation pulses at each defined threshold (sensory, motor, and maximal) with a 5–10 s break between each stimulation level. Recorded EMG signals were digitally sampled (2 kHz), amplified (gain 1000), and filtered (high pass Butterworth, 4th order, 30 Hz cutoff) during acquisition. Student EMG response data to tibial nerve stimulation and the different stimulation thresholds were collated and averaged to report the proposed method effectiveness.

#### Part 2: natural muscle recruitment

**Fig. 2:**
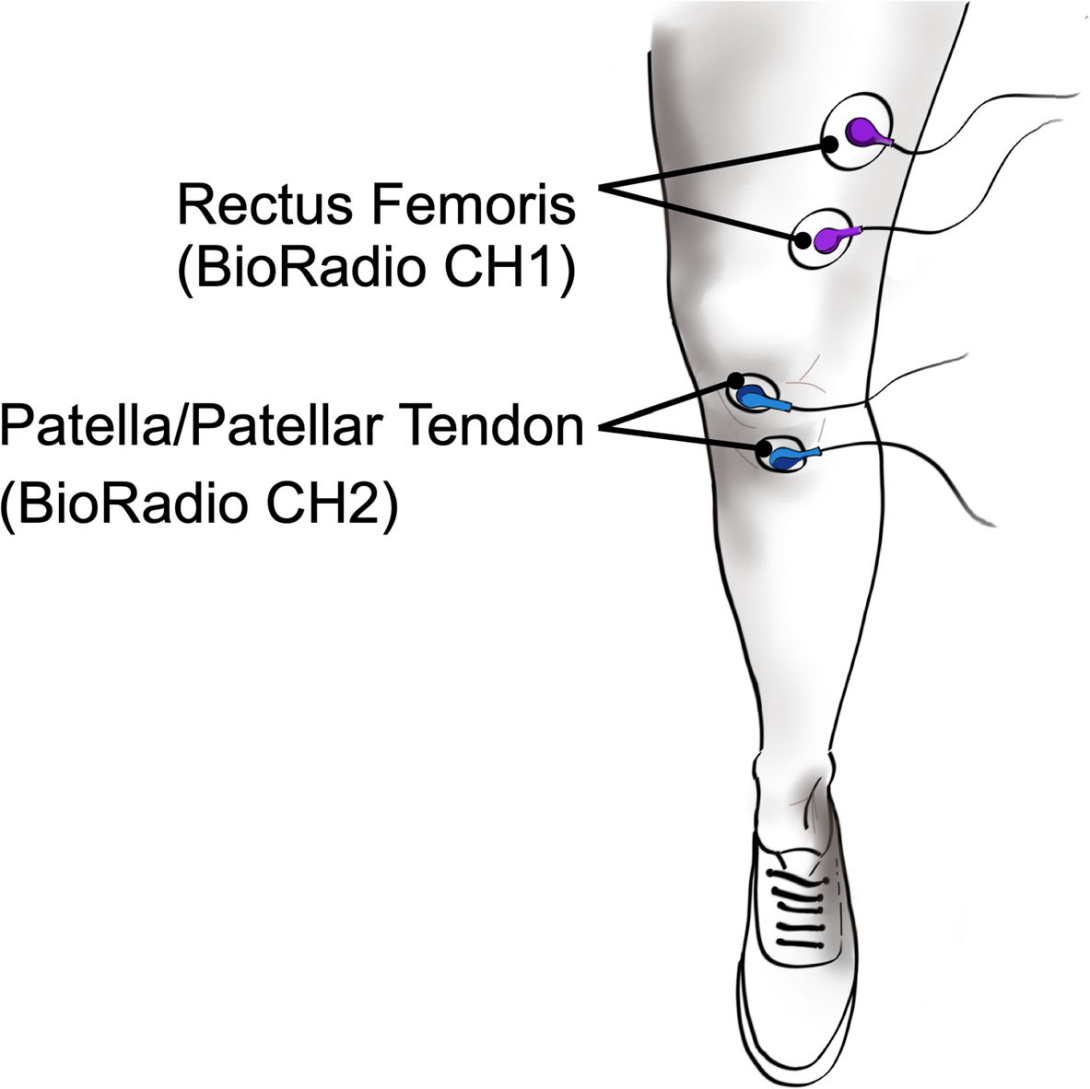
Placement of Recording Electrodes to Measure the Stretch Reflex Response. Two electrodes were placed longitudinally to measure contractions of the rectus femoris using the BioRadio (CH1). The other electrodes were connected to channel 2 (CH2) of the BioRadio and were place on the patella and patellar tendon. These electrodes were placed for detection of the tendon-tap that elicited the muscle contraction

EMG activity associated with the involuntary and naturally occurring knee-jerk response was collected. Again, an alcohol wipe was used to prepare the skin for electrode application above the rectus femoris, patella, and patellar tendon (Fig. 2). Two wet snap electrodes were placed longitudinally along the rectus femoris to measure the EMG response to the patellar reflex. Specifically, students were instructed to place the first electrode approximately four finger-widths proximal to the patella and the second placed two finger-widths above the first. Another pair of electrodes was placed on the patellar tendon and patella, *respectively,* to capture the hammer-strike as a signal artifact. All sensing and reference electrodes were connected to their respective two channels on the BioRadio and both signals were grounded with a lead connected to the lateral epicondyle.

Once electrodes were properly placed, the participants placed their knee joint at a 90-degree angle and relaxed their leg muscles. Prior to data collection, students practiced eliciting a knee-jerk response on the target subject. They also verified that a signal spike associate with the hammer strike and the respective rectus femoris contraction were observed in BioCapture. Upon signal verification, students then collected the EMG response to two different movements: the natural stretch reflex and the stretch reflex with the Jendrassik maneuver. Students performed each movement between 5 and 7 times with an approximately 5-s break between plexor taps and 10-s break between movements. As with the previous section, student EMG response to the different movements and the hammer stimulus data were collated and averaged as an indicator of the method success.

### EMG signal processing and analysis

All acquired signals were exported as comma separated value files and imported into MATLAB2015b (The MathWorks Inc., Natick, Massachusetts) for analysis.

#### Part 1: TENS-EMG

All EMG responses were epoched into 2 s windows centered around the TENS pulse artifact. Next, epoched data at the different stimulation thresholds (i.e., sensory threshold, motor threshold, and maximum tolerable threshold) were averaged for each student and the baseline deflection points were identified. The latency was then calculated as the peak-to-peak difference between the stimulus artifact and the subsequent wave response that corresponded to the muscle twitch. The twitch response amplitude was also measured and defined as the absolute maximum peak from the baseline (0 mV).

#### Part 2: stretch reflex

EMG signals were high pass filtered to remove motion artifacts (Butterworth, 6th order, cut off frequency 20 Hz), rectified, and subsequently low pass filtered (Butterworth, 4th order, cut off frequency 100 Hz) in order to remove high frequency noise. EMG data was epoched into 2 s windows centered around the temporal peak of the plexor artifact. The epoched EMG and hammer signals were each averaged for the natural and Jendrassik reflex trials for each student. Response latency was measured as the difference between the stimulus onset and positive peak corresponding to the rectus femoris contraction. Amplitude was also defined as the maximum voltage of the rectified signal from the baseline.

Statistical analysis was conducted using JMP Software (version 14.1, SAS Institute Inc., Cary, NC). The Wilcoxon Signed-Rank test was used to compare amplitude and latency data in response to different stimulation levels or stretch reflex movements. All values are reported and plotted as mean ± SEM and a *p*-value < 0.05 was considered significant.

### Survey collection and analysis

A survey instrument was created to assess student perceptions of this newly developed practical experience. Participation was advertised to students during lecture 1 week before the lab experience, and the survey was administered to participants by a teaching assistant. All data was anonymized and collated for assessment by a research volunteer not associated with data collection or the course.

Survey questions related to the specific objectives of the lab experience were used for the analysis and questions were grouped accordingly (Table 1; See ‘Additional file 1’ for Survey Instrument). The objectives were defined as: 1) how much the lab supported students in learning the course material; 2) to teach the students how to use the BioRadio and MATLAB for biosignal acquisition and analysis; and 3) to create a satisfying experience that promotes self-perception of the individual’s engineering skills.

**Table 1:** Survey Questions Relating to Each Lab Objective

Objectives	Survey Questions
Obj. 1: Supported Learning Physiological Concepts	• How would you rate the effectiveness with which course concepts were explained in Lab 2, the EMG lab exercise? • How would you rate the extent to which this laboratory experience improved your understanding of important course concepts related to skeletal muscle lectures? • How would you rate your confidence in the related subjects after completing this laboratory experience? • How would you rate the contribution of this laboratory experience to the value of your learning in the BME350H1 course (specifically, learning related to the muscular system)?
Obj. 2: Using the BioRadio and MATLAB for Biosignals	• How would you rate your confidence in your BioRadio EMG data acquisition skills after completing this laboratory experience? • How would you rate your confidence in application of your coding skills after completing this laboratory experience? • How would you rate your comprehension of EMG signal processing after completing this laboratory experience?
Obj. 3: Self-perception and Satisfaction	• How would you predict your comfort level in navigating this laboratory experience independently (if you did not have a partner)? • How would you rate the ease at which you were able to navigate through this exercise? • How would you rate the interactivity and your level of engagement with the laboratory experience? • How would you rate your critical thinking and evaluation skills after completing this laboratory experience?

Table 1 outlines the primary laboratory objectives and the survey questions used to qualitatively assess the students’ opinion on the experience. The survey questions were grouped under the relevant objectives outlined above.

Students responded to each question by selecting ‘very poor’, ‘poor’, ‘okay’, ‘good’, or ‘excellent’. A research volunteer not associated with the data collection coded the ordinal responses provided by each student on a 1–5 scale *respectively*. The student responses to the questions were grouped into the three categories as shown in Table 1. All responses for each category were used to generate a distribution for each objective. Next, the objectives were compared to determine whether there was a significant difference in the response distributions. Pair-wise comparisons between objectives (Obj1XObj2, Obj1XObj3, Obj2XObj3) was accomplished using a Pearson Chi-squared test in JMP. A *p*-value less than 0.05 was considered significant. To determine the average response for each objective, the mean ± SEM all the student responses in each category was calculated. To determine a general response to the lab for each student, the mean ± SEM response to survey questions 1–11 for each student was calculated. The response to Question 12 (‘In general, how would you rate this type of laboratory experience as an effective way to learn?’) of the survey was used to provide the general opinion of the lab among students (expressed as a percentage of the number of students who selected the same ordinal response over the total number of students).

## Results

### TENS EMG response

**Fig. 3:**
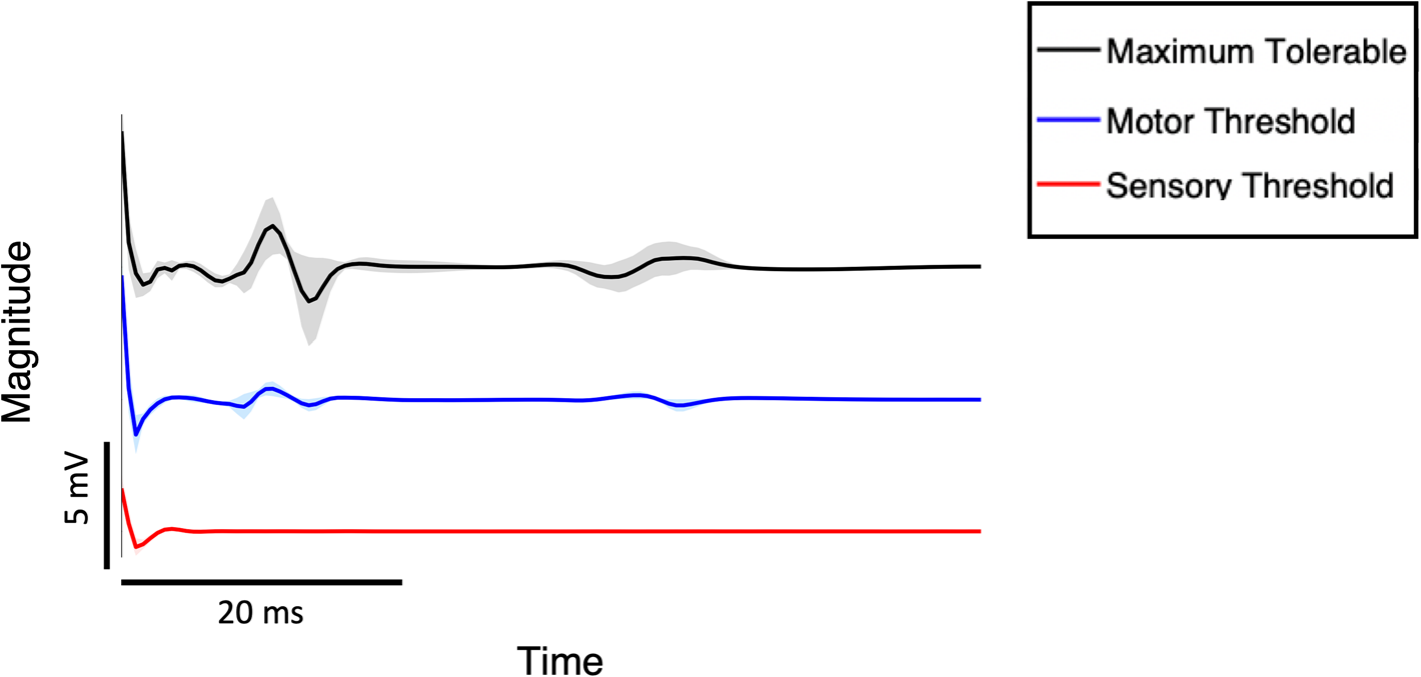
Electromyographic Response to Electrical Simulation of the Tibial Nerve. Plot representing the average Soleus muscle response at the sensory, motor, and maximum tolerable stimulation intensities. At the sensory threshold, no H-reflex was observed. The motor threshold evoked an M-wave in all 8 students and three participants also elicited an F-wave response at the maximum tolerable thresholds. With increasing stimulation intensity, there was a visibly larger amplitude response. Shaded regions represent ± SEM

The EMG response to electrical stimulation using the TENS device was collected in eight student participants. Students stimulated the tibial nerve several times while attempting to elicit different muscle responses at the sensory (total *n* = 103), motor (*n* = 142), and maximum tolerable (*n* = 148) intensities (Fig. 3). At the sensory threshold, no H-reflex was observed in any participants.

At the motor and maximum tolerable stimulation thresholds, EMG responses were observed in all students. At both threshold levels, an M-wave was elicited in all students approximately 13.2 ± 0.53 ms after the applied stimulation. There was a significant difference in the amplitude of the M-wave response (Wilcoxon signed-rank; Z = − 3.31; *p* < 0.01) at both intensities, with the maximum tolerable amplitude response (2.74 ± 1.40 mV) being over three times that at the motor threshold (0.87 ± 0.52 mV).

While a M-wave response was observed in all students, only three were able to evoke an F-wave approximately 39.3 ± 0.43 ms after stimulation (mean absolute amplitude 1.56 ± 0.33 mV; Fig. 3).

### Stretch reflex EMG response

**Fig. 4:**
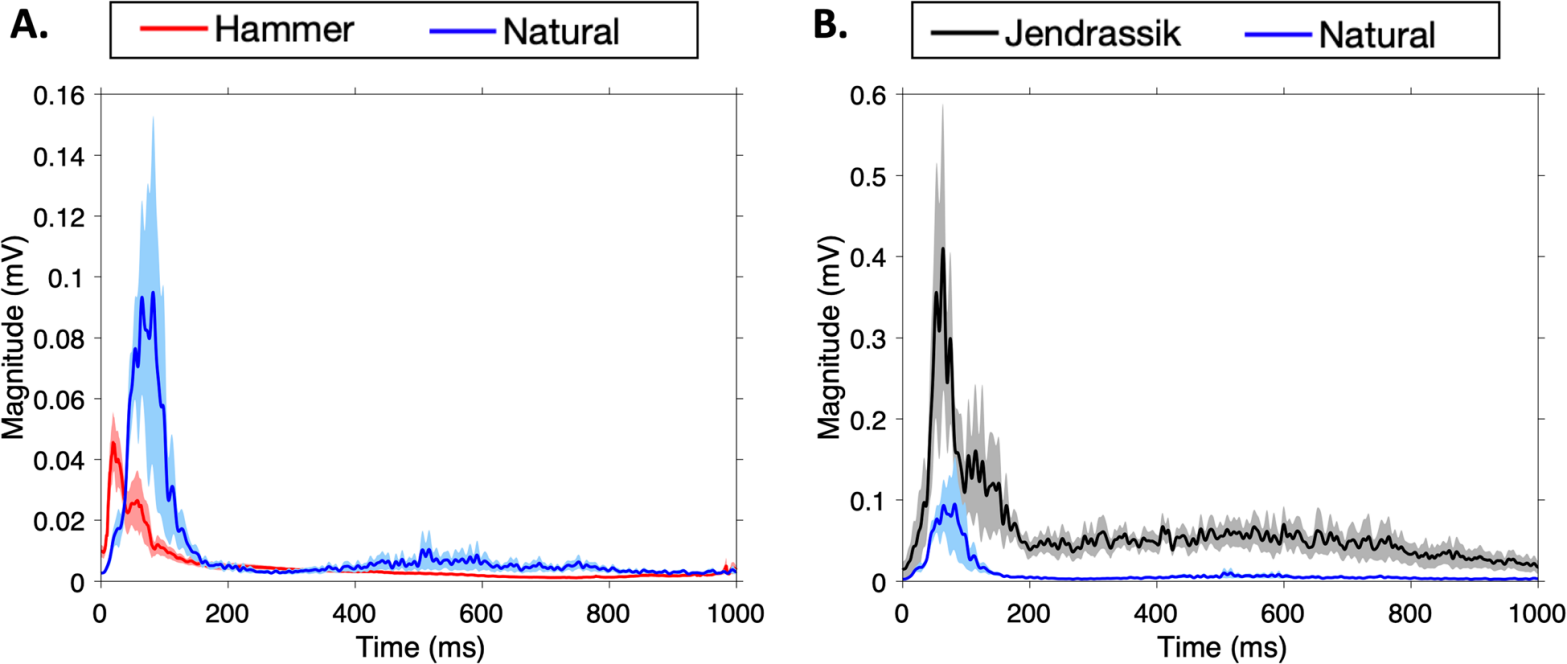
Electromyographic Response to the Patellar Hammer Strike. **a** Hammer (red) and EMG response (blue) signals were both measured during data collection. Due to electrode proximity and equipment limitations, it is possible that the EMG signal bled into the measured stimulus. However, a distinct difference is observed between the hammer strike onset and peak EMG response. **b** Aligning the EMG peak reflex response reveals that the Jendrassik maneuver caused a visibly larger response than that of the natural stretch reflex. Shaded regions represent ± SEM

Stretch reflex data from 6/8 students was used for this analysis. One dataset was removed from the analysis because the Jendrassik EMG signal was contaminated with the hammer artifact, making the two signals indistinguishable from one another. Another dataset was removed due to data corruption during acquisition. The remaining 6 student datasets were used for subsequent methods analysis (Fig. 4a).

As expected, the Jendrassik amplitude (0.50 ± 0.17 mV) response was significantly (Z = − 2.00; *p* < 0.05) larger than in the natural reflex (0.16 ± 0.06 mV) as shown in Fig. 4b. The latency did not differ significantly between the responses (Z = − 0.4; *p* = 0.69). However, the natural reflex was marginally more responsive (56.7 ± 3.8 ms) compared to the Jendrassik maneuver (60.7 ± 3.3 ms).

### Student survey feedback

**Fig. 5:**
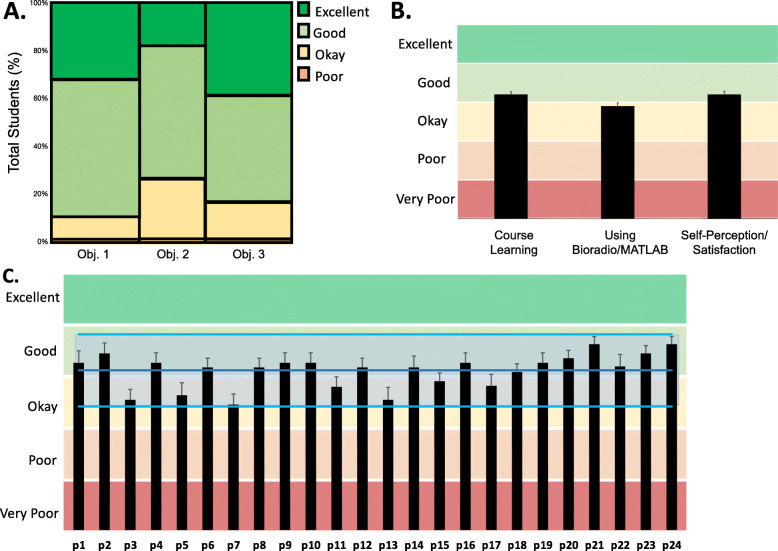
Class Response to Laboratory Experience Based on the Student Survey Instrument. **a** A contingency plot aggregates survey questions into three categories related to the primary objectives provided by this lab. Specifically, questions assessed how the lab helped students learn and apply course content, assessed effectiveness in teaching students how to use specific equipment (BioRadio/MATLAB) for biosignal acquisition and processing as well as defined student engagement; **b** The average student response (± SEM) for each objective shows the class was ‘okay’ or ‘good’ for all three objectives; **c** The mean ± SEM response of each student (represented as p#) for the questionnaire (regardless of category). The shaded blue region indicates the global mean ± StdDev for the class. This data shows most of the students reported ‘good’ for their questionnaire response; however, three students (p3, p7, and p13) felt the experience to be “okay”

A total of 24 students (100%) completed the exit survey on their lab experience with questions related to how well the lab supported course theoretical comprehension (specifically, neurophysiological concepts), improved their ability to acquire and analyze biosignals using the BioRadio system and MATLAB, as well as fostered application of their engineering skills. Most students rated ‘good’ or ‘excellent’ for all questions related to the three categories (Fig. 5a). For a general opinion on the lab as an effective way to learn, 23 of 24 students indicated that this new lab protocol was ‘good’ (45.8%) or ‘excellent’ (50.0%).

Pearson chi-square analysis revealed a significant difference between the ‘Self-perception/Satisfaction’ and ‘Course Learning’ categories compared to the ‘Using Bioradio/MATLAB’ category, (*χ*^2^ = 9.50; *p* = 0.02 *and χ*^2^ = 8.84; *p* = 0.03 *respectively*). The average response for ‘Course Learning’ (mean coded response 4.21 ± 0.07) and ‘Self-perception/Satisfaction’ (mean coded response 4.21 ± 0.07) was ‘good’ in comparison to ‘Using the BioRadio/MATLAB’ (mean coded response 3.90 ± 0.08) which leaned more towards ‘okay’. The differences in the distributions are attributed to more students stating ‘good’ or ‘excellent’ in response to Obj. 1 compared to Obj. 2 (total count 86 vs. 53). Whereas when comparing the responses to Obj. 2 versus Obj. 3, over half the students provided an ‘okay’ rating compared to ‘excellent’, *respectively* (total count 18 vs. 37). The majority of students indicated that the lab was ‘good’ (55.6%) or ‘okay’ (25.0%) in teaching use of the required lab equipment.

When looking at each individual student’s average response regardless of category, there were three students who rated significantly lower (average 3.54 ± 0.11) compared to the rest of the class (average 4.24 ± 0.04; *χ*^2^ = 41.99; *p* < 0.01).

## Discussion

In this study, we describe a new BME laboratory experience that integrates neural engineering and neurophysiological concepts to explore involuntary muscle reflexes using affordable, commercially available devices. Students exploited an affordable TENS device, the common patellar stretch reflex, and BioRadio Wireless Physiological Monitoring systems to acquire EMG signals. This protocol brings other unique elements to the sensorimotor learning experience for our students compared to many teaching methods described in the literature. First, students are required to capture biosignals from muscles in the lower extremities involved in the common patellar stretch reflex, a target not commonly used in teaching protocols likely due to the preferred ease of access to musculature in the face or upper extremities [14, 15]. By targeting the lower extremities in this protocol, we could explore involuntary reflexes that are induced by electrical stimulation (due to the superficial nature of the tibial nerve) and compare this response to the naturally induced stretch reflex. It is a unique feature of this protocol for students to apply an external source of stimulation to induce contractile activity and observe the reflexively-induced response. Generally, existing protocols include isometric or self-induced activation of contraction [14–16]. Additionally, signal processing using different software environments such as MATLAB has become an important part of data analysis. To provide greater challenge, this protocol requires our BME students to transfer acquired data from one software platform (BioCapture) to another (MATLAB) and effectively utilize this second program to further examine the influence of signal processing on data outcomes. Using different software platforms affords students the opportunity to learn that different approaches to interpreting data can be done with different platforms. Collectively, this makes a unique neurophysiological practical experience that allows BME students to compare physiological outcomes to theoretical concepts, make practical biosignal measurements, deal with biological variability, and design processing elements.

### Eliciting physiological responses

TENS is an inexpensive, nonpharmacological intervention that is commonly used for pain management [17]. Although there are a multitude of putative physiological mechanisms that explain the effects of TENS treatment, its core mechanism relies on triggering compound action potentials through short electrical stimulation pulses delivered by bipolar electrodes. Electrical stimulation pulses, such as those delivered by TENS devices, are ideal for studying nerve physiology because they provide a controllable stimulus for exciting peripheral nerves [18–21].

For the TENS-EMG portion of the described laboratory protocol, we focused on eliciting the Hoffman reflex (measured as an H-reflex), M-wave, and F-wave to teach BME students specific components of nerve physiology that correspond to these observed signals [21]. We targeted the tibial nerve because of its superficial nature in the popliteal space which makes it more susceptible to transcutaneous stimulation and easy for novice subjects to identify/define. EMG activity was measured in the soleus muscle because it is a readily accessible muscle directly below the skin’s surface that is innervated by the tibial branch. Furthermore, this muscle is commonly used to assess H-reflexes thereby providing an established comparison point for students during their analysis [12]. During execution of the current protocol, all students consistently excited the tibial nerve and observed a corresponding physiological response in the EMG signal.

At the sensory threshold the Hoffman reflex can occur during stimulation; however, none of our students elicited this response. Depending on the stimulation amplitude and an individual’s nerve activation threshold, motor neurons can be recruited by stimulating sensory neurons to initiate a response analogous to the natural stretch reflex (longer latency, appears as an H-reflex in the EMG) [12]. This response is caused by action potentials that travel orthodromically along afferent nerve fibers which synapse with alpha motor neurons in the spinal cord. The motor neurons subsequently activate efferent fibers that ultimately cause a synchronized muscle twitch that can be detected using EMG [12]. We suspect students did not observe an H-reflex due to various circumstances. Firstly, we were limited to transcutaneous stimulation even though H-reflexes are typically elicited using a percutaneous approach [12]. Additionally, improper electrode placement or body positioning may have diminished the observed response. A previous study demonstrated that individuals in a supine or prone position resulted in consistent H-reflex measurements; however, due to space constraints, our students were unable to assume such positions [12]. Furthermore, previous reports recommend stimulating the tibial nerve once every 10-s for a reliable response [12, 20]. The lowest stimulation frequency for our TENS devices was 2 Hz making it possible that Ia afferent neurotransmitters were depleted during stimulation which would have diminished the Hoffman response [19].

At the motor and maximum stimulation thresholds, students observed M- and F-waves that also correspond to compound muscle action potentials. Contrary to H-waves, M-waves typically have a significantly lower latency because they do not traverse the spinal cord and are easier to elicit. These waveforms manifest due to orthodromically-activated efferent fibers by electrical stimulation at the motor threshold. We found that all students successfully elicited an M-wave at the motor and maximal stimulation thresholds with an average latency (13.2 ± 0.53 ms) that is consistent with previous reports [22]. Additionally, as expected, the M-wave response amplitude was positively correlated to the stimulation intensity suggesting that a higher intensity likely caused more pronounced motor fiber recruitment.

In testing this new protocol, some students successfully identified F-waves at the maximum tolerable stimulus intensity. The average F-wave latency for these students (39.3 ± 0.43 ms) was also concordant with earlier studies that reported a post-stimulus response at approximately 40 ms [22]. F-waves are larger latency compound muscle action potentials that are elicited by a large external stimulus, which leads to antidromic activation of the alpha motor neurons [18]. Antidromic depolarization reaches the motor neuron cell bodies in the spinal cord and a small portion of these neurons “rebound” causing subsequent orthodromic activation of the target muscle. The corresponding muscle twitch is smaller than that caused by the initial orthodromic motor neuron activation (i.e., by the M-wave). However, not all students observed an F-wave and this may be attributed to activation threshold variability or, again, to incorrect electrode placement. It is also possible that participants were more conservative with their maximum tolerable intensity due to instructor emphasis on the potential risks of inappropriate TENS device use, or to the unaccustomed visual foot twitch during motor stimulation. For these reasons, it is possible that these novice users did not observe an F-wave because they were below the supramaximal stimulation threshold needed to evoke the response [22].

This protocol also required students to implement the knee-jerk response as a mechanism to learn about naturally occurring stretch reflexes. Measuring the EMG response after the tendon-tap allowed students to investigate peripheral nerve conduction velocities and learn about the clinical utility of the stretch reflex. Students successfully acquired EMG signals in response to plexor strikes performed during the knee-jerk response. The average stretch reflex latency was 56.7 ± 3.8 ms which is larger than that reported by Pope and colleagues (~ 35 ms) [23]. This discrepancy can be explained by our analytical approach where we defined latency as the difference between the onset of the hammer strike to the EMG peak response, rather than the onset. In addition to eliciting the natural stretch reflex, students also sensitized the response with the Jendrassik maneuver. As expected, the EMG amplitude was significantly larger when the students performed the Jendrassik maneuver compared to the natural stretch reflex.

### Student response

The response distributions between the different survey categories related to the lab experience showed that, overall, the lab was effective in meeting the three primary objectives. Based on the student response, the laboratory protocol was particularly effective in supporting the lecture material and engaging students’ engineering skills because the average response was ‘good’. Students were given the option at the end of the survey to provide any additional comments or thoughts on the lab. One student reflected “Because the lab deliverables aren’t hard or demanding, the focus is on learning which is nice”. The lab was weaker in teaching students how to use the BioRadio/MATLAB for biosignal acquisition and processing; however, it must be noted that the average response was still relatively positive with most students responding ‘okay’ or ‘good’ for that objective. The challenge could be attributed to the novelty of the BioRadio and minimal experience processing biosignals in MATLAB because, at this stage in their education (3rd year undergraduate), these students have not been required to take any signal processing courses in their program. These attributes are supported by individual comments in which students suggest the knowledge gap stemmed from insufficient exposure to electrical signal acquisition and the theory behind signal processing approaches. As one student commented, the “lectures focused mostly on [physiological] mechanisms and less on electrical signals. More info on the math behind signal processing would be nice”. Another student expressed that they wish they had more experience with the equipment when saying “Not to ask for more work but having more practice could reinforce things”. Despite these limitations, the disconnect associated with no prior BioRadio/MATLAB experience did not detract from the overall interest in the laboratory experience and learning about neurophysiology. Additionally, it must be considered that the undergraduate students in the BME cohort had diverse backgrounds and interests. This course included students who study tissue engineering along with those interested in the robotic or medical device aspects of our multidisciplinary field. A difference in individual interest on the lab topic could explain the particularly lower rating for three students compared to the rest of the class. Yet even with this variation among students, most reported the overall experience as ‘good’ or ‘excellent’ which speaks to the meaning of the lab as a supplement to the course. One student commented “The labs are an interactive and fun aspect of the course!” and another indicated that “The experience was great”. This laboratory exercise provided an opportunity for students to directly interface with the peripheral nervous system and explore different methods to evoke involuntary reflexes. Survey responses indicated that the students overall found the lab engaging and an interactive approach to learning about neurophysiology.

### Suggested improvements

While the general student response was largely positive, there are several avenues for improving this laboratory exercise. We recommend that as part of their BME coursework, the students should be provided a brief overview of MATLAB and relevant biosignal processing methods. During physiology course lectures, instructors should also review electrical signals that can be transduced and measured to learn about the BioRadio system. Furthermore, since our students were unable to elicit the H-reflex, stimulating at the sensory threshold added limited value to their experience and therefore this step may be unnecessary in the context of this practicum. It may be fruitful to remove this component, change the student’s posture (supine or prone as opposed to seated), or stimulation protocol (low amplitude, < 0.1 Hz) to increase the chances eliciting the Hoffman reflex.

## Conclusion

We determined that this newly developed laboratory protocol provided a feasible and engaging experience for our third-year undergraduate BME students to directly integrate involuntary reflex physiology and basic neural engineering concepts. We demonstrated that the BioRadio physiological monitoring system, used in conjunction with an affordable TENS device, can evoke and measure appropriate physiological responses. Furthermore, this multifaceted experience not only provided students with the opportunity to actively apply their physiological course concepts but also introduced biosignal processing, which may be useful to them as future engineers. The exercise required students to use MATLAB which could increase student programming literacy and better prepare them for a future in industry or academia. This protocol provides a reliable and robust framework for other undergraduate BME programs to actively teach neuromuscular physiology, biological signal acquisition, and neural engineering.

## Supplementary Information

**Additional file 1.** This file is the survey instrument that was used to guage the student response to the lab. The file shows the questions students were asked regarding the lab experience.

**Additional file 2.** This is the laboratory protocol given to the students. It provides more detail on the data acquisition and analysis process.

## Data Availability

The datasets used and/or analyzed during the current study are available from the corresponding author on reasonable request.

## Abbreviations

BME: Biomedical Engineering
CH1: Channel 1
CH2: Channel 2
EMG: Electromyography
FES: Functional Electrical Stimulation
NMES: Neuromuscular Electrical Stimulation
TENS: Transcutaneous Electrical Stimulation
