# Supplementary material for: A biomedical Engineering Laboratory module for exploring involuntary muscle reflexes using Electromyography
Source: J Biol Eng. 2020 Nov 9;14:26. doi: 10.1186/s13036-020-00248-z (PMC7650172; doi:10.1186/s13036-020-00248-z)
Supplement: Supplementary file 1 — Additional file 1. This file is the survey instrument that was used to guage the student response to the lab. The file shows the questions students were asked regarding the lab experience. [file 13036_2020_248_MOESM1_ESM.pdf]

BME350H1 Lab 2 EMG Survey Questions

1. How would you rate the effectiveness with which course concepts were explained in Lab 2, the EMG lab exercise?

A. Excellent
B. Good
C. Okay
D. Poor
E. Very poor

2. How would you rate the extent to which this laboratory experience improved your understanding of important course concepts related to the skeletal muscle lectures?

A. Excellent
B. Good
C. Okay
D. Poor
E. Very poor

3. How would you predict your comfort level in navigating this laboratory experience independently (if you did not have a partner)?

A. Excellent
B. Good
C. Okay
D. Poor
E. Very poor

4. How would you rate the ease at which you were able to navigate through this laboratory experience?

A. Excellent
B. Good
C. Okay
D. Poor
E. Very poor

5. How would you rate the interactivity and your level of engagement with the laboratory experience?

A. Excellent
B. Good
C. Okay
D. Poor
E. Very poor

6. How would you rate your confidence in the related subjects after completing this laboratory experience?

A. Excellent
B. Good
C. Okay
D. Poor
E. Very poor

7. How would you rate your confidence in your BioRadio EMG data acquisition skills after completing this laboratory experience?

A. Excellent
B. Good
C. Okay
D. Poor
E. Very poor

8. How would you rate your confidence in application of your coding skills after completing this laboratory experience?

A. Excellent
B. Good
C. Okay
D. Poor
E. Very poor

9. How would you rate your comprehension of EMG signal processing after completing this laboratory experience?

A. Excellent
B. Good
C. Okay
D. Poor
E. Very poor

10. How would you rate your critical thinking and evaluation skills after completing this laboratory experience?

A. Excellent
B. Good
C. Okay
D. Poor
E. Very poor

11. How would you rate the contribution of this laboratory experience to the value of your learning in the BME350H1 course (specifically, learning related to the muscular system)?

A. Excellent
B. Good
C. Okay
D. Poor
E. Very poor

12. In general, how would you rate this type of laboratory experience as an effective way to learn?

A. Excellent
B. Good
C. Okay
D. Poor
E. Very poor

13. Do you have any other comments or suggestions about this laboratory experience?
