# Supplementary material for: A biomedical Engineering Laboratory module for exploring involuntary muscle reflexes using Electromyography
Source: J Biol Eng. 2020 Nov 9;14:26. doi: 10.1186/s13036-020-00248-z (PMC7650172; doi:10.1186/s13036-020-00248-z)
Supplement: Supplementary file 2 — Additional file 2. This is the laboratory protocol given to the students. It provides more detail on the data acquisition and analysis process. [file 13036_2020_248_MOESM2_ESM.pdf]

Introduction

Origin of the Electromyography (EMG Signal)

Skeletal muscle refers to muscle cells that are attached to bones and provide voluntary limb movements. These somatic muscles are attached to bone by tendons, therefore a muscle contraction causes the tendon to pull on the bone and this generates movement. Skeletal muscles are made up of many muscle fibers and the strength of the contraction is dependent on the number of fibers that are synchronously contracting with a given muscle. Each muscle fiber is innervated by a single motor neuron, but a single neuron can innervate upwards of several hundred muscle fibers. A motor neuron and all fibers that it innervates is referred to as a 'motor unit'. The number of muscle fibers innervated by a single neuron is called the innervation ratio. A lower innervation ratio corresponds to finer control of muscle forces.

Action potentials (AP) via the motor neuron cause synchronous fiber activation and is the origin of a muscle contraction. APs have a duration of approximately 1-3 ms and result in muscle contractions, or 'twitches', that last approximately 10-100 ms.

Electromyography (EMG) records the collective electrical activity produced by muscles. EMG can be measured through surface electrodes (which we will use during this lab) and through a percutaneous approach (needle electrodes inserted directly into the muscle). A disadvantage of surface EMG is that it can only measure superficial muscles and detects activity over a large area making it more susceptible to crosstalk. The EMG signal is a summation of the signal produced by many muscle fibers at the same time. The single motor units do not fire synchronously. The AP produced by each fiber has both positive and negative components. Therefore, summation of the evoked field potentials of all the active motor units at the skin surface produces a waveform that is essentially random. Although the signal may appear random, the muscle force output can be related to the amplitude of the recorded EMG signal. Additionally, the amplitude in the time domain is related to the number, size, and frequency of recruited motor units.

Neuron Fiber Types

The peripheral nervous system primarily consists of nerve bundles, which are bundles of neuronal axons that have cell bodies either in the peripheries, or in the spinal cord. These nerve bundles consist of both efferent (i.e. going away from the central nervous system) and afferent (i.e. going towards the central nervous system) fibers, which allow the brain and spinal cord to send voluntary and/or autonomic commands to and receive sensory input from the body respectively. Axons for different neurons have different diameters and the conduction velocity (i.e. the speed at which an action potential can travel down an axon) varies depending on the diameter of the fiber. Similar to an electrical cable, axons with larger diameters have higher conduction velocities. Sensory neurons tend to have a large diversity of axonal diameters. Sensory neurons that carry mechano-sensory information tend to have medium and large diameter axons (i.e. types I/Aα and II/Aβ fibers), whereas a majority of sensory neurons that carry information from pain and temperature receptors have small diameter non-myelinated

fibers (i.e. type IV/C fibers). On the other hand, motor neurons in the periphery (typically α motor neurons) tend to have large diameter myelinated axons.

The Electroneurogram (Reflexly Evoked Field Potentials)

In this lab we will be electrically stimulating the tibial nerve which is a peripheral nerve that originates within the L4-S3 spinals segments and innervates the back/side of the leg from the knee to the foot. It provides motor input to several deep and superficial muscles of the leg. We will be measuring the motor unit recruitment of the soleus muscle which controls ankle flexion.

As mentioned in the previous section, some peripheral nerves, like the tibial nerve, are "mixed" in that they are composed of both sensory (afferent) fibers and motor (efferent) fibers. Physiological activation of nerves is very different from electrical activation. Under physiological conditions, the body recruits the smallest diameter fibers first then the largest. Additionally, sensory fiber activation transmits APs from the peripheral limbs to the central nervous system (CNS) while motor fibers convey information from the CNS to the peripheral muscles. Electrical stimulation using a transcutaneous electrode nerve stimulator (TENS) elicits the opposite effect: it can activate both sensory and motor nerve fibers simultaneously. Additionally, it recruits fibers from the largest diameter to the smallest.

When a mixed peripheral nerve is electrically stimulated and a contraction is recorded in the muscle it supplies, it typically elicits a distinct EMG response**.** At **low stimulation intensities (i.e. low amplitude)**, large diameter nerve fibers are stimulated because they have the lowest recruitment threshold. These large diameter fibers are sensory fibers that conduct towards the CNS and ultimately synapse with motor fibers that elicit a contraction in the muscle target.

This muscle response is known as the H-reflex. At a **medium intensity,** small diameter motor fibers are recruited along with large diameter sensory fibers. This causes a sort latency muscle contraction called the M-wave which is caused by direct activation of the motor fibers. This is followed by the slower latency H-wave response. With **high intensity stimulation**, more motor fibers are recruited and the refractory period of these fibers interferes with the H-wave so only the large amplitude M-wave is produced.

Supramaximal nerve stimulation eliminates the H-wave and instead elicits the F-wave response. The F-wave is similar to the H-reflex in that it occurs after the M-wave however, it is caused by **antidromic** activation of motor fibers rather than activation of the reflex arc. In this situation, the evoked action potential travels in the reverse direction along the motor fiber to the CNS rather than to the target muscle. When the stimulus reaches the spine, it depolarizes the cell and causes an action potential to fire and activate the target muscle. The F-wave has a small amplitude (0.2-0.5 mV).

Measuring the muscle response to evoked electrical stimulation of a peripheral nerve allows us to characterize the latency and nerve conduction velocity. These measurements can assess peripheral nerve function and help diagnose neuromuscular disorders.

Voluntary Control of Movement

Voluntary control of movement is typically initiated in the primary motor cortex (M1) which is a region of the frontal lobe of the cerebral cortex, just anterior to the central sulcus. This region of the brain has motor neurons called upper motor neurons that directly connect to lower motor neurons through the lateral corticospinal tract (which is a tract of fibers that travels down the spinal cord). The upper motor neurons (in conjunction with other motor areas such as the premotor and supplementary motor areas) are involved in initiating the movement signal. The signal is then passed onto lower motor neurons, which are the neurons that innervate skeletal muscles in order to execute the voluntary movement. The movement signal initiated by the primary motor cortex can be processed and refined by the cerebellum and can be further processed at the level of the spinal cord. Voluntarily initiating movement in response to a sensory stimulus is a relatively complex process. It requires the sensory information to travel through afferent sensory fibers, up the spinal cord to the brain, where it gets processed in various cortical regions. Eventually, the processed sensory stimuli cause activation of upper motor neurons in the part of the motor cortex corresponding to the part of the body that is intended to move. The signal must then travel down the spinal cord, through the lower motor neurons and then to the skeletal muscles to complete the movement. As you can imagine, it takes quite some time for the signal to travel this distance, and every time the signal must be transferred from one neuron to another through a chemical synapse, the latency of the signal increases. Therefore, this high latency is not suitable for postural reflexes, such as the stretch reflex, which are discussed below.

Involuntary Control of Movement (Spinal Reflexes)

When a motor response to a sensory stimuli is required at a low latency, spinal reflexes are involved. As the name suggests, spinal reflexes are mediated by neurons in the spinal cord. The simplest of these reflexes is the stretch reflex, in which a muscle (or group of muscles) respond to a sudden stretch in the muscle (or the associated tendon) with a rapid contraction. The most common example of a stretch reflex is the knee-jerk reflex. In the knee jerk reflex, a tap of the patellar tendon causes a rapid increase in the length of rectus femoris muscle. This sudden increase in the length of the biceps femoris muscle is sensed by muscle spindles, which initiate action potentials in the corresponding sensory neurons (which typically have type I axon diameters). These sensory neurons have direct excitatory synapses on the motor neurons of the same muscle (in this case, the rectus femoris muscle) and in turn, cause a contraction of this muscle. The same sensory neurons also have excitatory synapses on interneurons within the spinal cord, which in turn inhibit the antagonist muscle group (in this case, the biceps femoris muscle, also known as the hamstring) in order to allow for the joint to move (in this case, for the knee to open). Stretch reflexes are also amongst the lowest latency reflexes, in part because the sensory information travels through the largest diameter fibers! While most reflexes are self-sufficient (i.e., they don’t require any input from higher level centers in the brain), most can be modulated by some form of supra-spinal control. In the case of stretch reflexes, individuals can decrease the amplitude of the reflex if they consciously anticipate and try to inhibit movement. To overcome such supra-spinal control and to properly elicit stretch reflexes, sometimes clinicians use distraction methods such as the **Jendrassik Maneuver** in which individuals are asked to voluntarily clench their teeth or lock their fingers together and pull apart as the stretch

reflex is elicited. Doing so lifts the volitional inhibition of the reflex and generally elicits a larger amplitude spinal reflex.

Learning Objectives

1. Examine EMG signals using a Bioradio
2. Discern the difference in latency between involuntary and voluntary muscle contractions
3. Recruit motor unit activity using a TENS device
4. Calculate nerve conduction velocities

Materials

1. Partner
2. Bioradio Kit (6 snap electrodes and snap leads)
3. Laptop
4. TENS device with electrodes
5. Reflex hammer

Safety Information

Transcutaneous electrode nerve simulating (TENS) devices electrically activate nerves and muscles by delivering current to the tissue. **TENS devices are FDA approved and safe to use.** They are commonly used in a clinical and home settings to treat a variety of pain and muscular disorders. **HOWEVER, if used improperly, they can result in serious injury or permanent damage to your body.** Please take the following precautions during this lab:

- If you are uncomfortable using a TENS device or are unable to for any reason, please talk to the TA to be paired with someone who is willing/able to use the device.
- **WHEN STIMULATING, START AT THE LOWEST STIMULATION AMPLITUDE.** Gradually increase the amplitude at your comfort level to elicit an effect. If you start to feel pain, do not continue to increase the amplitude; either decrease to a comfortable level or stop stimulation.
- Never use a TENS device on atrophied muscles, muscles with spasms, muscles associated with an impaired joint or limb, or undiagnosed painful muscles.
- Do not use the TENS device if you have a cardiac pacemaker, defibrillator, or other implanted metallic or electronic devices.
- Do not place the electrodes on or near the heart or chest or neck because induction of electrical current can cause rhythm disturbances in the heart or spasm of laryngeal or pharyngeal muscle can occur constricting airways.
- Do not apply electrodes on open wounds or inflamed, swollen, or infected skin areas.
- Do not move electrodes while the TENS power is ‘on’.
- The points above are most relevant to our lab and the design studio environment. For more safety information please refer to the following manual:

  https://bodyclock.co.uk/content/manuals/Classic-TENS.pdf

Disclaimer: Please remember that the data you collect using the BioRadio kit is intended for experimental discussion only. This data is not intended to be used to assess your actual health and does not substitute for analysis by your physician. Do not use this equipment in conjunction with a defibrillator. Reconsider equipment use if you are, or may be, pregnant. Be aware that improper routing of leads may result in a choking hazard.

Part 1: Data Collection

1.1 Muscle Fiber Recruitment with TENS

1. Prepare your skin for the surface electrodes by cleaning your soleus muscle area and back of the knee (Popliteal space) using an alcohol wipe.
2. Take 3 snap electrodes and connect the snap leads to them. The electrodes will measure the EMG activity of your soleus muscle.
3. Place the reference and sensing electrodes on the soleus and connect these electrodes to CH1+ and CH1- of your Bioradio). Place the ground electrode on your ankle bone (*medial malleolus*) and connect to the GND port on your Bioradio.
4. Turn on your Bioradio (ensure your Bioradio is disconnected from the computer).
5. Turn on your laptop and launch the BioCapture Software. Click Device→Select Device. When the menu that opens, select the radio that corresponds to the label on your BioRadio. Click Connect.
6. Once the software has located your device, click “Device Config”.
7. Ensure that the **Differential** option is selected under Programmable Channels. Set the sample rate to 2 kHz, only tick the checkbox next to CH1 and under Type, select “EMG” and label the channel as EMG. Ensure that no other sensors or channels are selected. Click **Program Device** in order to exit back to the main screen.
8. At the top of the tool bar, click the triangle icon, corresponding to “Start Acquisition”.
9. Adjust your “Graph Timespan” to 10s.
10. Contract and relax your soleus muscle a few times (you can do this by flexing your calves) and observe the signal. If you do not see a signal, try adjusting your y-axis by right-clicking on the axis→select ‘Y-Axis→click ‘Auto Scale Quick’. If you still do not see a signal, verify the electrodes are connected and placed properly and the Bioradio is on. If you are still having trouble, ask your TA.
11. If your EMG signal is appropriate, stop the acquisition (by clicking the square icon) and **switch off your Bioradio**.
12. Take two stimulating electrodes and place them at the back of your knee on top of the tibial nerve. Confirm with your TA to ensure you have proper placement.
13. Connect the electrodes to the TENS device cable. **DO NOT CONNECT IT TO THE TENS DEVICE YET.**
14. **Before connecting the cable to the TENS, verify that the amplitude is set to 0. This means that the device is off. If it is on, switch the device off. ENSURE THAT THE CATHODE (i.e. the RED LEAD) connects to the electrode that is more proximal (i.e. farther up your leg), and the ANODE (i.e. the black lead) connects to the electrode that is more distal (i.e. farther down your leg).**
15. Connect the electrode cable to TENS device
16. Open the front of the device by sliding down the top panel. On the B-C-M or B-N-M switch, set the device to ‘C’ or ‘N’ depending on your TENS device model.

17. Set the frequency to 2 Hz and pulse width to 250 µs. **What would this stimulus waveform look like?**
18. Slowly increase the amplitude of the stimulator until you can feel a sensory stimulus, but there is no observable muscle twitch. **Note this amplitude** (this is often called the **sensory threshold**). Now further increase the amplitude of the stimulator until you see an observable muscle contraction in the calf muscles. **Note this amplitude** (this is often called the **motor threshold**).
19. Turn off the stimulator and turn on the Bioradio. Reconnect your Bioradio to the BioCapture software.
20. Click 'Run'. Slowly increase the amplitude of the stimulator. You will notice a large amplitude stimulation artifact. You may notice activity after the artifact. This is the recruitment of muscle fibers through stimulation! We can tell it is greater that the motor threshold because there are F-waves. At the motor threshold we would only see M- waves. Below the motor-threshold there would only be the stimulus artifact and (perhaps) an H- reflex.
21. Increase the amplitude to the maximum tolerable amplitude and record this amplitude (**Tmax)**. You might even be able to tolerate the maximum amplitude (level 5 or 10 depending on which stimulator you have). Turn the stimulator off.
22. If the system is setup and functioning properly, you are ready to collect the data.
23. Click 'Record' and save the file as '*TENS'*. Once you save the file, BioCapture will automatically start recording.
24. Turn the stimulator on and quickly set the amplitude to the **sensory threshold** and allow for ~5-7 pulses.
25. Turn the stimulator off for 5 seconds. Continue recording.
26. Turn the stimulator on again but this time, quickly set it the amplitude to the **motor threshold (Tmot)**. Allow for ~5-7 pulses.
27. Turn the stimulator off for 5 seconds. Continue recording.
28. Turn the stimulator on again, but this time, quickly set the amplitude to the **maximum tolerable amplitude (Tmax)**. Allow for ~5-7 pulses.
29. Stop recording and acquisition by clicking the square icon.

1.2 Measure Reflex Arcs

1. Remove the stimulating and recording electrodes from your leg.
2. Locate the **rectus femoris** muscle on your thighs. It is the largest quadricep muscle that runs down the front, center of your thigh and prepare the skin overlying this muscle for surface electrodes by wiping with alcohol swabs.
3. Take two snap electrodes and place them longitudinally on the rectus femoris muscle. Place one of them ~4 finger widths above the top of the patella and the other one ~2 finger widths above that one.
4. Take another snap electrode and place it on the bony part of your elbow. This will be the grounding electrode.
5. Connect the snap leads to your electrodes and the leads to CH1+/- and grounding electrode to GND.
6. Place your knee joint at approximately 90-degree angle and relax your leg muscles. Let your foot hang in the air. Locate the patellar tendon by palpating for the patella (i.e. the kneecap) and then palpate just below it. You should feel a hard yet compressible structure which connects the patella to the tibial bone. This is the patellar tendon. Take the reflex hammer and have your partner gentle hit your patellar tendon to induce an involuntary reflex.
7. Take two electrodes and place one on your patella and about an inch below place the other on the patellar tendon. Connect the electrode leads to CH2+/-. These electrodeswill measure the motion artifact of the reflex hammer.
8. Ensure that your **BioRadio** is turned on and connected to the BioCapture Software.
9. Click the "Device Config" option. Ensure that the sampling rate is set to **2kHz**. Check only CH1

and CH2 and make sure that both recording modes are set to **differential.** Label CH1 as EMG and select 'EMG' as type. Label CH2 as hammer, select the type as 'custom', and set the voltage range to 2 µV. Click 'Program Device'.

10. At the top of the tool bar, click "run" and adjust your Graph Timespan to 10s.
11. Contract and relax your rectus femoris muscle (quadricep muscles) a few times and adjust the CH1 y-axis as before to ensure that you can see the voluntary muscle contractions.
12. Hit the patellar tendon with the reflex hammer (as close to the patellar tendon electrode as possible) to induce an automatic reflex. On the CH2 plot you should see a sharp spike that corresponds to the hammer hitting the knee. Adjust the Y-axis as needed.
13. During the recording, you will induce three movements: involuntary reflex and involuntary reflex with the Jendrassik maneuver.
14. Before recording, practice each of the movement until you and your partner are confident to perform them.
    - **Involuntary reflex:** as before have your partner gently hit your knee with the reflex hammer to induce involuntary movement
    - **Involuntary reflex with Jendrassik maneuver:** Lock your fingers together. Your partner will say 'Go' right as they are about to strike the tendon. At this cue, pull your fingers as hard as you can as your partner taps the patellar tendon.
15. Once you're comfortable with doing these movements, start the recording. Save the file as 'Reflex' move onto the next step. When you click save, BioCapture will automatically start recording.
16. Wait ~5 seconds. Have your partner strike your tendon ~5-7 times to induce an involuntary reflex.
17. Wait ~10 s. Have your partner strike your tendon ~5-7 times to induce an involuntary reflex while you perform the Jendrassik maneuver.
18. When you are finished with the recording, click on the record button again 'red dot' to stop the recording.
19. Turn off and remove the Bioradio.

Part 2: Export Data from Biocapture

1. In BioCapture click File→Open→Recording... and select your *TENS* file.
2. Click Data→Export to .CSV File... and then save the file as *TENS.csv*. Be sure to export the filtered data and use 'Tab' as the delimiter. Save the file to your desktop.
3. Repeat with your 'Reflex' data.

Part 3: Analyze data in Matlab

3.1 Muscle Fiber Recruitment with TENS

1. Open MATLAB and click Import Data in the toolbar. Select the *TENS*.csv file. This will open a data import menu. In this menu, select the column corresponding to CH1 and select all the rows except for the first one (which corresponds to the title of the row). Be sure to import as a 'Column Vector' and then click 'Import'.
2. Create a time vector corresponding to the EMG signal using (t = 0:N-1 /fs). Where N is the length of your TENS vector, and fs is your sampling rate.
3. Plot the time against the recorded EMG signal (Ch1).
4. Zoom in to the evoked contractions and observe if there are any M-waves or F-waves at the lowest

amplitude (sensory threshold). **Why might you not see any activity?** Take a screenshot of the best recorded waveform, copy it and paste it into a PowerPoint file and label. If you do not see any evoked response, record the M, and F amplitude as 0.

5. If you see an evoked response, measure the latency and amplitude of the response and record these values in your excel sheet. For latency, use the ginput() function to measure the time corresponding to the peak of the stimulus artifact and the time point of the evoked EMG response (This will be the time where the EMG signal first deflects from the baseline recording). Latency will then be calculated as:

   Latency = evokedResponseTime – stimulusArtifactTime

   The amplitude of your evoked response will be:

   Amplitude = evokedResponseMaxVoltage – baselineVoltage
6. Again, use the ginput() function to determine the evoked response of your signal. If you see a response at the sensory threshold, this will correspond to the H-wave.
7. If you do not see an evoked response, set the H-, M-, and F-waves latencies and amplitudes to 0.
8. Do the same with the remaining levels (Motor Threshold and Maximal Threshold). At some point, you will start to see the M-wave and then the F-wave.
9. When you see an M- or F-wave (any evoked response) measure and record its latency and amplitude using the ginput() function.
10. With the motor threshold stimulation, take a screenshot of your best evoked response and save it to your PowerPoint file. **What was the average latency of your M-wave?**
11. For the maximum threshold stimulation, take a screenshot of your best evoked response and save it to your PowerPoint file. **What was the average latency of your F-wave?**
12. Plot the stimulus intensity versus the wave amplitude for the H, M, and F waves (include all on the same plot). **Do you notice any patterns?** Save the plot to your PowerPoint.
13. Determine the mean H-, M-, and F- wave latencies for your subject.
14. Using a tape measure, measure the limb length of the subject's leg from the mid popliteal line to the medial malleolus. Record the limb measurement and subject's height. Later we will look at the data from your classmates to see if there is a correlation between limb length/height and latency. Record these measurements in your PowerPoint

3.2 Measuring Reflex Arcs

1. Clear all your variables and close all figures in Matlab. Click the Import Data button in the toolbar. Select the *Reflex.csv* file that you previously saved. In the opened import data window, select columns that contain the EMG data and the Reflex Hammer data (don't include the 'time column). On the top toolbar, ensure the data will be imported as column vectors. Make sure that you don't import the first row of the file since this has the column labels. Click 'Import Selection'.
2. In your workspace, you should have 2 variables: EMG and Hammer.
3. Plot the EMG and Hammer signals against time on the same plot using 'hold on'. **Do you see any patterns or latency differences between the signals?**
4. Find and save the onset time of each of the patellar strike by clicking on each peak on your Hammer signal with the ginput() function.
5. Use the ginput() function to find the onset time of the EMG reflex response by clicking at the start of the reponse (which is the point that the signal first deflects from the baseline). Measure the onset and amplitude of the EMG response.
6. Compare the average reflex amplitude between the three movements. **Do you notice any changes in the amplitudes? Why might this be?**

7. Subtract the EMG onset to its corresponding hammer strike time to calculate the latency. Average the latency for each of the three movements. **Do you notice differences in the latencies? Why does the voluntary contraction have a longer latency that the involuntary reflex?**
